# Mathematical analysis of the dynamics of cyberattack propagation in IoT networks

**DOI:** 10.1371/journal.pone.0322391

**Published:** 2025-05-16

**Authors:** Yousef AbuHour, Sadeq Damrah, Mahmoud H. DarAssi, Zuhur Alqahtani, Areej Almuneef

**Affiliations:** 1 Department of Basic Sciences, Princess Sumaya University for Technology, Amman, Jordan; 2 Department of Mathematics and Physics, College of Engineering Australian University, West Mishref, Safat, Kuwait; 3 Department of Mathematical Science, College of Science, Princess Nourah bint Abdulrahman University, Riyadh, Saudi Arabia; Cardiff Metropolitan University - Llandaff Campus, UNITED KINGDOM OF GREAT BRITAIN AND NORTHERN IRELAND

## Abstract

The growing threat of cyberattacks is a severe concern to governments, military organizations, and industries, especially with the increasing use of Internet of Things (IoT) devices. To tackle this issue, researchers are working on ways to predict and prevent these attacks by studying how malware spreads. In this study, we use a discrete-time approach to better model how cyberattacks spread across IoT networks. We also focus on the role of firewalls, developing a strategy to optimize their effectiveness in slowing down the spread of malware. Additionally, we analyze the reproduction number’s sensitivity and explore the proposed discrete system’s local and global stability. The model was simulated and analyzed using Python packages, providing practical solutions to improve cybersecurity in IoT networks. These insights are supported by numerical simulations based on real-world data.

## 1 Introduction and literature review

DDoS attacks utilize several compromised computer systems as attack-traffic sources. DDoS attacks may result in financial loss, intellectual property theft, database destruction, and reputation damage, among other malicious activities [1, 2]. Cyber security studies allow professionals to counter such attacks to avoid disruption in critical services and ensure national security. Knowing how different DDoS attacks work is key to providing the right defense against them. Various strategies for mitigating DDoS attacks exist to ensure security against such an attack [3]. In summary, the study of cyber and DDoS attacks cannot be ruled out in modern times in light of the need to understand the threats and develop protective measures to ensure the continuous operation of core services and foster national security.

Several mathematical models can be used to simulate DDoS attacks over IoT networks. One of these mathematical models is the decision tree-based IDS proposed by Kumar *et al*. [4]. This model avoids intra-network and inter-network DoS/DDoS attacks in the IoT environment. The authors have used a virtual machine with the Cooja simulator, which was pre-installed in the Contiki operating system for conducting experiments. The results showed that the C5 Decision Tree-Based IDS model was very accurate, with low rates of false alarms [4]. In this line, Oluwafemi *et al*. [5] compared the effectiveness of supervised, unsupervised, and semi-supervised machine learning algorithms on detecting DDoS attacks in Cyber-Physical Systems IoT (CPS-IoT). Their dataset consisted of 10,000 records for training and testing the algorithms in their case. Results showed that a supervised machine learning algorithm best detects DDoS attacks [5]. Al-Sarawi [6] also simulated and analyzed different DDoS attack scenarios using different attack rates and buffer sizes of network components. They used Omnet++ to simulate the DDoS attacks and assess the impact on the performance of a network. Researchers and cybersecurity experts can use these models to understand how DDoS attacks work and build a strategy to counter them. The mathematical modeling of the DDoS attacks shall help understand their behavior for developing suitable countermeasures. Machine learning can be used to model DDoS attacks in IoT networks. A DDoS attack will consume all of the resources of a victim’s server, making it unable to accept connections from new clients [7, 8].

SIR models in epidemiology are primarily employed in studying the dynamics of the spread of infectious diseases. Recently, some researchers have started to use SIR models to study the spread of cyberattacks. It helps prevent attacks by detecting network vulnerabilities and predicting attack propagation. The dynamics of the propagation of diseases and the control strategies were relatively explored in works [9–11] by researchers. They have used systems of differential equations and developed mathematical frameworks to simulate transmission dynamics under various scenarios, from strict social restrictions to the application of optimal control measures in transmitting different diseases. These models consider differential equations that deal with population behavior to understand how such behavior affects the spread of a disease, thereby establishing the effectiveness of interventions. On the other hand, the research uses age-structured models to express the dynamics of diseases with differential equations and study the efficiency of vaccination strategies. Introducing impulsive vaccination in the above model, they ante the time and optimal coverage to regulate the spread [12–14]. Though having a different theme, their work on the burden of diseases and their mitigation also resorts to differential equations in modeling the impact of mitigation strategies for diseases. For example, to cope with drug resistance, it combines education intervention with a "test and treat" strategy in a differential equation framework to project its potential for surmounting such drug resistance. In particular, across these studies, the mathematical framework of differential equations becomes a powerful tool for understanding and controlling the dynamic spread of diseases.

The modeling of the spreading of an attack in a network confounds which nodes are more vulnerable and thus take measures to protect them [15, 16]. Models have earlier been built to simulate cyberattacks so that the enthronement of the defense strategies and prevention of the results could be lessened. The researchers used artificial intelligence in the detection and prediction of cyberattacks. Researchers have put forward research, including Pokhrel *et al*. [17], on bots concerning security-related issues.

In the work of [18], the authors devised a trust-based hypothetical scheme based on a mathematical model, classifying the devices into different categories by analyzing their internal behavior through the calculated trust score.

Microsoft Security Blog that provides insights into DDoS attack trends in 2022. According to the report, Microsoft mitigated an average of 1,435 attacks daily in 2022. The maximum number of attacks per day recorded was 2,215 on September 22, 2022. The minimum number of daily attacks was 680 on August 22, 2022. Table 1 describes the approximate number of DDoS attacks and the average cost of those attacks in years 2020, 2021, and 2023.

**Table 1:** The number of DDoS attacks and the average cost of attacks.

Year	2020	2021	2022
Number of DDoS Attacks	10,089,687	9.75 million	1,435 attacks per day
Average cost of DDoS attack	$132,000	$174,000	$218,000

In a report published by Infosecurity Magazine, the frequency of DDoS attacks increased by 74% year-over-year in 2022. Nevertheless, the rate of growth started to decelerate in the fourth quarter, with attacks dropping by 53% in December. The power of botnets continued to grow throughout the year, making possible attacks greater than 2 Tbit/s and persisting for as long as three days [19, 20]. Some of these data are from the report in Table 2.

**Table 2:** The number of bots and the source of botnet DDoS attacks in 2022.

Botnet source	China	United States	India
Number of bots	590,000	376,000	350,000

In recent research on modeling malware spread over IoT networks, much of the related work has primarily focused on continuous-time differential equation systems. While these models provide valuable insights, they often lack the granularity to capture real physical systems’ discrete nature. Our research addresses this gap by adopting a discrete-time modulation approach, which offers a more accurate representation of the step-wise progression of malware spread within IoT environments. Unlike previous studies, we also incorporate the efficiency of firewalls into our model, introducing an optimal control strategy that dynamically adjusts firewall effectiveness to minimize malware propagation. Additionally, our work delves into the sensitivity of the reproduction number, highlighting its critical role in determining the threshold conditions for successful malware containment. This comprehensive approach bridges the gap between continuous and discrete modeling and provides actionable insights for enhancing cybersecurity measures in IoT networks.

This study aims to develop a mathematical model for simulating cyberattacks, specifically DDoS attacks, on IoT devices. The objectives of this study are as follows: To identify vulnerabilities in IoT networks through the proposed mathematical modeling of cyberattacks DDoS on IoT devices. To predict the spread of an attack and determine the most vulnerable nodes. To provide recommendations to decision-makers for new policies to protect networks from future attacks. Determine the loss caused by cyberattacks and the cost of protection. We determined the cost of protection from these attacks to model the variables related to cyberattacks. The workflow of this study is as follows: The dynamic behavior of cyberattack propagation was simulated using a system of differential equations. The stability of the proposed model was analyzed by calculating the basic reproduction number to measure the attack’s impact. This study investigates the influential parameters and relationships between the factors that affect the spread of an attack. The number of malicious IoT devices that influence attacks and vulnerable and protected devices was estimated and predicted. The results from the proposed model are leveraged to set optimal control strategies for specific parameters, thereby reducing the cost of prevention and losses caused by cyberattacks. Finally, the study analyzed the attacking space devices and the number of malicious and suspicious devices.

## 2 Methodology

In this work, we can assume that the targeted IoT space consists of all IoT devices connected within a specific network, which can be divided into two types based on their security protection level. Also, it is commonly known that IoT devices are vulnerable to cyberattacks primarily due to their lack of built-in security features, weak or hard-coded passwords, unpatched vulnerabilities, and inadequate security solutions. Additionally, many users do not change the default settings on their devices, making them even more vulnerable. As a result, attackers can quickly gain access to private networks and steal sensitive information [21]. To raise the security level of IoT devices, it is crucial to implement security solutions such as firewalls, antivirus, and encryption. Additionally, devices should be updated regularly to patch security vulnerabilities, and strong, unique passwords should be used for each device. As an additional layer of protection, blockchain technology can be used to secure IoT devices further. Therefore, let *S*_*a*_ denote the set of vulnerable IoT devices, and *S*_*p*_ represent the set of protected devices. The notation of targeted IoT devices that become malicious is *M*_*t*_.

**Fig 1:**
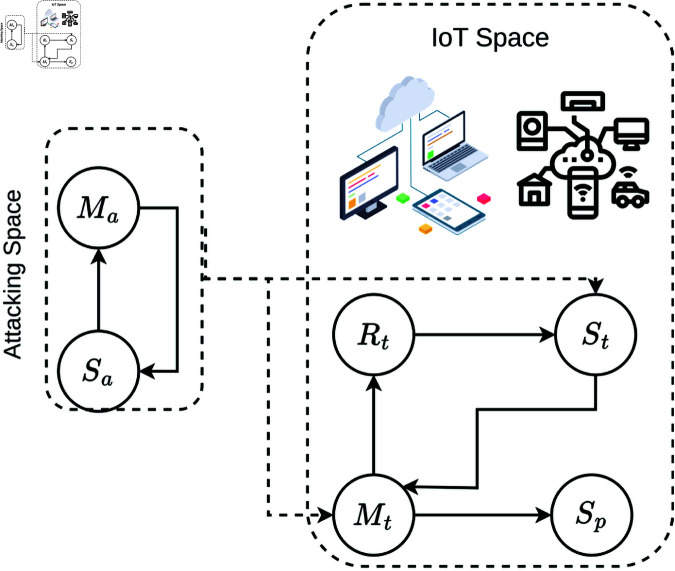
Illustration of model compartment links.

The mathematical framework models the attack dynamics through four key scenarios. In the first scenario, attackers use compromised devices (*M*_*a*_) to spread malicious code, launch DDoS attacks, or gain access to sensitive data and other devices on the network. This behavior is captured by the interaction terms $\beta_{a}S_{a}M_{a}$ and $\beta_{t}S_{t}M_{t}$ in the equations. In the second scenario, compromised target devices (*M*_*t*_) transition to a protected state (*S*_*p*_) at a rate *p*, as described by ${\dot{S}}_{p}=pM_{t}$. The third scenario involves the recovery of compromised devices (*M*_*t*_), which move to a recovered state (*R*_*t*_) at a rate $\sigma_{t}$, modeled by ${\dot{R}}_{t}=\sigma_{t}M_{t}-\theta_{t}R_{t}$. Finally, in the fourth scenario, recovered devices (*R*_*t*_) can become susceptible again at a rate $\theta_{t}$, reintroducing them into the vulnerable pool. The four scenarios are illustrated in Fig 1, which visually represents the transitions and interactions between the compartments

Attackers’ space, attacked devices are defined as follows:

$${\dot{S}}_{a}=-\beta_{a}S_{a}M_{a}-\theta_{a}S_{a}+\nu_{a}M_{a},$$ (1)

$${\dot{M}}_{a}=\beta_{a}S_{a}M_{a}-\theta_{a}M_{a}-\nu_{a}M_{a}.$$ (2)

Target population targeted IoT devices are modeled as follows:

$${\dot{S}}_{t}=\Pi -\lambda \;S_{t},\;\dot{M_{t}}=\lambda S_{t}-\sigma_{t}M_{t}-pM_{t},\;{\dot{R}}_{t}=\sigma_{t}M_{t}-\theta_{t}R_{t}\;{\dot{S}}_{p}=pM_{t}$$ (3)

where $\lambda =\beta_{a}M_{a}+\beta_{t}M_{t}$ and $𝒩=S_{a}+M_{a}+S_{t}+M_{t}+R_{t}+S_{p}$

Table 3 presents the model parameters along with their corresponding values used in the simulation and analysis of the results.

**Table 3:** The description of the system’s parameters (Fig 1).

Parameter	Description	Values and units
Π	The arrival rate of new IoT devices into the susceptible population.	10 devices/h (assumed)
$\beta_{a}$	Effective contact rate for attacking space.	0.5 packets/device/h (assumed)
$\theta_{a}$	The failure rate of attacked IoT devices is naturally included, such as power depletion losses.	0.1 h^−1^ (assumed)
$\sigma_{a}$	Rate of recovered malicious IoT devices.	0.2 h^−1^ (assumed)
$\beta_{t}$	Effective contact rate for the targeted population.	0.3 packets/device/h [22]
$\nu_{a}$	The recovery rate of attacking population.	0.05 h^−1^
$\sigma_{t}$	Rate of recovered malicious IoT devices for target space.	0.2 h^−1^ [23]
$\theta_{t}$	The failure rate of targeted IoT devices is naturally included, such as power depletion losses.	0.1 h^−1^ [22]
*p*	The protection rate, transferring vulnerable IoT devices to secure protected.	0.1 h^−1^ (assumed)

## 3 Model formulation and analysis

This section presents a mathematical model for DDoS attack propagation in IoT networks, adaptable to other attack types with necessary modifications.

Botnets, like Mirai and Hajime, are designed to infect vulnerable IoT devices, creating large networks of compromised devices (*M*_*a*_ and *M*_*t*_) that can coordinate DDoS attacks. The model’s emphasis on infection, recovery, and protection directly reflects the behavior of botnets in real-world scenarios, making them a natural fit for the system’s dynamics. Table 4 categorizes various types of malware commonly employed in Distributed Denial of Service (DDoS) attacks targeting Internet of Things (IoT) devices [24, 25]. Botnets represent the most suitable type of malware for the given model because they align with the dynamics of infection, recovery, and protection described in the equations. The model captures the spread of malware in both the attacker’s network (*S*_*a*_ and *M*_*a*_) and the target network (*S*_*t*_ and *M*_*t*_), including infection rates ($\beta_{a}$ and $\beta_{t}$), recovery rates ($\nu_{a}$ and $\sigma_{t}$), and protection mechanisms (*p*).

**Table 4:** Malware types used in DDoS attacks on IoT devices.

Malware type	Example	Role in DDoS attacks
Botnets	Mirai, Hajime, Bashlite	Primary tool for DDoS attacks; infects IoT devices to form large networks for coordinated assaults.
Worms	Reaper (IoTroop)	Spreads rapidly to vulnerable devices, expanding botnets used in DDoS attacks.
Trojans	LightAidra (Aidra variant)	install backdoors, enabling remote control of devices as part of DDoS botnets.
Rootkits	Firmware Rootkits	Hides botnet malware, ensuring devices remain compromised for long-term DDoS participation.
Fileless Malware	Memory-resident malware	Operate stealthily in device memory, temporarily hijacking devices for DDoS without leaving traces. Less common in IoT devices.

The discrete-time system model for analyzing the spread of malware in IoT networks. The model consists of two interconnected populations: the attacker population and the target population.


$$S_{a}^{n+1}-S_{a}^{n}=-\beta_{a}S_{a}^{n+1}M_{a}^{n+1}-\theta_{a}S_{a}^{n+1}+\nu_{a}\;M_{a}^{n+1}$$

$$M_{a}^{n+1}-M_{a}^{n}=\beta_{a}S_{a}^{n+1}M_{a}^{n+1}-(\theta_{a}+\nu_{a})\;M_{a}^{n+1}$$

and the targeted population is:

$$S_{t}^{n+1}-S_{t}^{n}=\Pi -\left(\beta_{a}M_{a}^{n+1}+\beta_{t}M_{t}^{n+1}\right)S_{t}^{n+1}$$ (4)

$$M_{t}^{n+1}-M_{t}^{n}=\left(\beta_{a}M_{t}^{n+1}+\beta_{t}M_{t}^{n+1}\right)S_{t}^{n+1}-\sigma_{t}M_{t}^{n+1}-pM_{t}^{n+1}$$

$$R_{t}^{n+1}-R_{t}^{n}=\sigma_{t}M_{t}^{n+1}-\theta_{t}R_{t}^{n+1}$$

$$S_{p}^{n+1}-S_{p}^{n}=pM_{t}^{n+1}$$

**Lemma 1.** For non-zero initial conditions, the solution for model (4) satisfies: $\lim_{k\to \infty }\sup{𝒩}^{k}\leq 1$

*Proof:* Upon using the fact that


$${𝒩}^{k+1}=S_{a}^{k+1}+M_{a}^{k+1}+S_{t}^{k+1}+M_{t}^{k+1}+R_{t}^{k+1}+S_{p}^{k+1}$$

$$={𝒩}^{k}+\Pi -\theta_{a}\left[S_{a}^{k+1}+M_{a}^{k+1}+R_{t}^{k+1}\right]$$

Since, $\theta_{a},S_{a},M_{a}$, and *R*_*a*_ are all positive quantities and Ω is an invariant region, then we may set Π=0, and hence we have


$${𝒩}^{k+1}\leq {𝒩}^{k}$$

Similarly, we can conclude that


$${𝒩}^{k}\leq {𝒩}^{k-1}\leq 𝒩(0),\;𝒩(0)={𝒩}^{0}=1$$

Hence, $\lim\sup_{k\to \infty }{𝒩}^{k}\leq \lim\sup_{k\to \infty }{𝒩}^{k-1}\leq \lim\sup_{k\to \infty }{𝒩}^{0}\leq 1$. Therefore, $\lim\sup_{k\to \infty }{𝒩}^{k+1}\leq 1$, and the proof is completed. ◻

Thus, Lemma 1 proved that the set $\Omega =\{\left(S_{a},M_{a},S_{t},M_{t},R_{t},S_{p}\right)\text{ s.t }N^{k}\leq 1\}$ is positively invariant.

## 4 Free malicious malware equilibrium point (FMME)

The FMME is the Free Malicious Malware Equilibrium Point, a conjectural point in cybersecurity where the population of malicious software becomes stable within a network. In this situation, the rate of new malware infection into the system is equated with the rate by which the same malware is detected and neutralized. In FMME, the overall effect of malware on the network remains constant, which indicates that current defense measures can significantly suppress such threats but never actually stamp out the problem. Knowing and identifying the FMME may help cybersecurity specialists optimize their strategies to ensure that resources are well allocated to maintain network stability and security.

### 4.1 Local stability analysis of the malware-free equilibrium

The Free Malicious Malware Equilibrium Point’s local stability refers to a system’s ability to regain equilibrium following minor perturbations. From a network perspective, this means that at FMME, although the increase or decrease of the malware infection rate may slightly influence all defenses, they will adjust to restore the balance. Moreover, local stability in its own right is instrumental in ensuring uniformity in security, where small changes in malware activity do not lead to large disruptive pixel changes. The FMME’s local stability would have to be framed against how fast detection and mitigation mechanisms react to and recover from the events. Cybersecurity designs should enhance this local stability to ensure that minor threats never become explosive security breaches. In this section, we will investigate the local stability of the FMME for the system (4). The steady state at the equilibrium point when an attack is not successful, “Vanished attack,” or free of malicious malware equilibrium point (FMME) is assumed to be ${ℰ}_{0}=\left(S_{a}^{*},M_{a}^{*},S_{t}^{*},M_{t}^{*},R_{t}^{*},S_{p}^{*}\right)=(1,0,1,0,0,0)$.

Calculating the basic reproduction number ${ℛ}_{0}$ will assure the stability of the model at this equilibrium point. Using the next-generation matrix approach as described by [26–30], the matrices ${ℳ}_{infection}$ and ${ℳ}_{transition}$ are evaluated as follows:


$${ℳ}_{infection}=[\begin{array}{cc} \beta_{a} & 0\\ \beta_{a} & \beta_{t} \end{array}],$$

and


$${ℳ}_{transition}=[\begin{array}{cc} \theta_{a}+\nu_{a} & 0\\ 0 & \sigma_{t}+p \end{array}]$$

According to [30],${ℛ}_{0}$ can be considered the spectral radius of the matrix $𝒳={ℳ}_{infection}\;{ℳ}_{transition}^{-1}$ i.e. ${ℛ}_{0}=\rho \left(𝒳\right)$.

It can be defined as the reproductive number, which is the extent of maximum damage expected from a cyberattack. It is the total reproductive numbers of the attacker population space and the target space. If the reproductive number of the attacking population crosses one, the nodes flowing from suspicious to malicious increase uncontrollably, resulting in a successful attack.


$${ℛ}_{0}=\max\{{ℛ}_{\text{attack}},\;{ℛ}_{\text{Target}}\}=\max\{{ℛ}_{0}^{a},\;{ℛ}_{0}^{t}\}$$

where


$${ℛ}_{\text{attack}}=\frac{\beta_{a}}{\theta_{a}+\nu_{a}}\;\text{and}\;{ℛ}_{\text{Target}}=\frac{\beta_{t}}{\sigma_{t}+p}$$

To put it another way, if the reproduction number is more significant than one, then each infected malicious node, on average, will successfully infect more than one further node, which causes an exponential increase in malicious nodes. This fast-spreading makes the attack hard to stop and dramatically raises the chances of success.

**Lemma 2.** The FMME point of the model (4), ${ℰ}_{0}=\left(S_{a}^{*},M_{a}^{*},S_{t}^{*},M_{t}^{*},R_{t}^{*},S_{p}^{*}\right)$, is locally asymptotically stable if ${ℛ}_{0}<1$, and unstable if ${ℛ}_{0}>1$.

*Proof:* The proof of this lemma can be deduced from [30] (see Theorem 2 and S1 Appendix A). ◻

### 4.2 Global stability of FMME

The global stability of a free malicious malware equilibrium in a cyberattack model means the system will asymptotically reach a malware-free state regardless of where it starts. This involves establishing that the malware-free equilibrium is globally stable, as demonstrated by any of the system’s trajectories resulting in an arbitrarily chosen initial state. The development of global stability requirements almost certainly involves introducing a suitable Lyapunov function, effectively measuring a system’s potential energy. Such a function should exhibit a monotonic decrease over time and suggest its energy dissipation capabilities, thus leading to a steady state. When the failure of the initial computer systems due to bugs is not considered, this generally becomes the description of what is called “convergence” and “equilibrium” in the industry. An equilibrium is supposed to be attained once the interim phase is over and the incoming malware is “cleaned” by the antivirus programs (or other methods). The crucial property that guarantees system behavior over the long term in terms of security and the absence of malicious threats that will damage the dignity of the network or system under consideration is thanks to this property.

**Theorem 1.** At the malware-free equilibrium point ${ℰ}_{0}$, the proposed model (4) is globally asym-ptotically stable whenever ${ℛ}_{0}\leq 1$.

*Proof:* Following the general procedure for the global stability [31–34], we consider the following Lyapunov function


$${ℱ}_{1}^{k}=c_{1}\;M_{a}^{k}+c_{2}\;M_{t}^{k}+c_{3}\;R_{t}^{k}$$

where


$$c_{1}=\frac{1}{\theta_{a}+\nu_{a}},\;c_{2}=\frac{1}{\sigma_{t}+p},\;\text{and}\;c_{3}=\frac{1}{\theta_{t}}.$$

The backward difference of ${ℱ}_{1}$ is given by

$$\Delta {ℱ}_{1}={ℱ}_{1}^{k+1}-{ℱ}_{1}^{k}$$ (5)

$$=\frac{1}{\theta_{a}+\nu_{a}}\left[M_{a}^{k+1}-M_{a}^{k}\right]+\frac{1}{\sigma_{t}+p}\left[M_{t}^{k+1}-M_{t}^{k}\right]+\frac{1}{\theta_{t}}\left[R_{t}^{k+1}-R_{t}^{k}\right]$$ (6)

$$=\frac{1}{\theta_{a}+\nu_{a}}\left[\beta_{a}S_{a}^{k+1}M_{a}^{k+1}-\left(\theta_{a}+\nu_{a}\right)M_{a}^{k+1}\right]$$ (7)

$$+\frac{1}{\sigma_{t}+p}\left[(\beta_{a}M_{a}^{k+1}+\beta_{t}M_{t}^{k+1})S_{t}-\left(\sigma_{t}+p\right)M_{t}^{k+1}\right]$$ (8)

$$+\frac{1}{\theta_{t}}\left[\sigma_{t}M_{t}^{k+1}-\theta_{t}R_{t}^{k+1}\right]$$ (9)

By the application of lemma 1, $S_{a}\leq 1$ and $S_{t}\leq 1$ in Ω. Thus, we obtain

$$≦\left[\frac{\beta_{a}}{\nu_{a}+\theta_{a}}-1\right]M_{a}^{k+1}+\left[\frac{\beta_{t}}{\sigma_{t}+p}-1\right]M_{t}^{k+1}$$ (10)

$$\left[{ℛ}_{0}^{a}-1\right]M_{a}^{k+1}+\left[{ℛ}_{0}^{t}-1\right]M_{t}^{k+1}$$ (11)

$$\Delta {ℱ}_{1}\leq 0\;\text{whenever}\;{ℛ}_{0}\leq 0.$$ (12)

Using the La Salle invariant principle [29], we conclude that the MMFE point ${ℰ}_{0}=(1,0,1,0,0,0)$ of the model (4) is global asymptotically stable (GAS) in Ω. ◻

## 5 Endemic equilibrium

The endemic equilibrium in the cyberattack model is a state in which a certain amount of malignant malware is retained in the system, yet it is constant. At this point, the rate of new infections is balanced by a recovery or neutralization rate, leading to a stable, though nonzero, malware prevalence. Knowing the endemic equilibrium is very important for estimating the long-term impact of cyber threats and mitigation strategies. The stability of such an equilibrium is analyzed to determine whether small perturbations will die out or lead to fluctuations in malware prevalence. The control at this point over the system dynamics is, therefore, very critical in maintaining cybersecurity to avert wide-range damages that arise from persistent cyber threats.

### 5.1 The endemic equilibrium point (EEP) existence

An EEP’s existence often relies on the basic reproduction number, ${ℛ}_{0}$. When ${ℛ}_{0}>1$, the EEP exists, meaning that infection can persist in a population. On this basis, one can analyze the conditions for the existence of an EEP to understand the settings under which malware persists and design appropriate control measures that will reduce the malware burden or eradicate it.

Let the endemic equilibrium point, where a long-term DDoS attack be

${ℰ}^{**}=\left(S_{a}^{**},M_{a}^{**},S_{t}^{**},M_{t}^{**},R_{t}^{**}\right)$. We can derive the following result.

**Lemma 3.** If $\beta_{a}>\nu_{a}$, the model (4) has a unique endemic equilibrium point ${ℰ}^{**}\in {\mathbb{R}}_{+}^{5}$ .

*Proof:* Substituting the expression for ${ℰ}^{**}$, into the model (4) at steady state yields


$$-\beta_{a}S_{a}^{**}M_{a}^{**}-\theta_{a}S_{a}^{**}+\nu_{a}M_{a}^{**}=0$$

$$\beta_{a}S_{a}^{**}M_{a}^{**}-\left(\theta_{a}+\nu_{a}\right)M_{a}^{**}=0$$

$$\Pi -\left[\beta_{a}M_{a}^{**}+\beta_{t}M_{t}^{**}\right]S_{t}^{**}=0$$

$$\left[\beta_{a}M_{a}^{**}+\beta_{t}M_{t}^{**}\right]S_{t}^{**}-(\sigma_{t}+p)M_{t}^{**}=0$$

$$\sigma_{t}M_{t}^{**}-\theta_{t}R_{t}^{**}=0$$

Regarding the attackers’ compartments, we have:


$$-\beta_{a}S_{a}^{**}M_{a}^{**}-\theta_{a}S_{a}^{**}+\nu_{a}M_{a}^{**}=0$$

$$\beta_{a}S_{a}^{**}M_{a}^{**}-\left(\theta_{a}+\nu_{a}\right)M_{a}^{**}=0$$

Upon solving the equations we obtain,

$$S_{a}^{**}=\frac{1}{R_{0}^{a}}$$ (13)

$$M_{a}^{**}=\frac{\theta_{a}}{-\beta_{a}+\nu_{0}R_{a}^{0}}$$ (14)

By solving the target compartments steady-state equations, we get:

$$M_{t}^{**}=\frac{\Pi }{\sigma_{t}+p}=\Pi \frac{R_{0}^{t}}{\beta_{t}}$$ (15)

$$S_{t}^{**}=\frac{\Pi R_{0}^{a}}{\beta_{a}+\Pi R_{0}^{t}R_{0}^{a}}$$ (16)

$$R_{t}^{**}=\frac{\sigma_{t}M_{t}^{**}}{\theta_{t}}=\frac{\sigma_{t}\Pi R_{0}^{t}}{\beta_{t}\theta_{t}}$$ (17)

Since all the parameters are positive, it implies that $S_{a}^{**}>0$, $S_{t}^{**}>0$, $M_{t}^{**}>0$ and $R_{t}^{**}>0$. However, $M_{a}^{**}>0$ only if $\beta_{a}>\nu_{a}$. That is, $M_{a}^{**}>0$ whenever $R_{0}^{a}>1$. ◻

### 5.2 The endemic equilibrium point (EEP) stability analysis

The stability of the (EEP) for cyberattacks becomes essential to understanding such systems’ long-term behavior. An EEP will indicate a steady state in which the system is coexisting with some constant level of cyber threats in the environment. Stability analysis answers whether small perturbations around this state will decay in time and, therefore, self-limit, returning the system to its equilibrium or growth, eventually leading to divergence from equilibrium. The local stability of the EEP is generally checked with different methods, among them the linearization and eigenvalue analysis of a system’s Jacobian matrix, evaluated at the EEP. If all of the eigenvalues have negative genuine parts, then the EEP will be locally asymptotically stable; that is, it will return to equilibrium after minor disturbances. Such stable behavior is essential in the development of robust strategies for cybersecurity, guaranteeing resilience to persistent threats and minimizing the risk of far-flung cyber incidents.

Define $\Omega_{0}\subset \Omega$ as the set where all compartments other than the suspicious ones go to zero. i.e.

$$\Omega_{0}=\{(S_{a},M_{a},S_{t},M_{t},R_{t})\in \Omega |M_{a}=S_{t}=M_{t}=R_{t}=0\}$$.

**Theorem 2.** If ${ℛ}_{0}>1$, the globally asymptotically stable (GAS) only one point (EEP) point of the model (4) exists within $\Omega /\Omega_{0}$.

*Proof:* Define the following Lyapunov function

$${ℱ}_{2}=\frac{1}{2}[S_{a}^{n+1}-S_{a}^{**}+M_{a}^{n+1}-M_{a}^{**}+S_{t}^{n+1}-S_{t}^{**}+M_{t}^{n+1}-M_{t}^{**}+R_{t}^{n+1}\;-R_{t}^{**}+S_{p}^{n+1}-S_{p}^{**}{]}^{2}$$ (18)

$$=\frac{1}{2}{\left[{𝒩}^{n+1}-{𝒩}^{**}\right]}^{2}$$ (19)

$$\Delta {ℱ}_{2}={ℱ}_{2}^{n+1}-{ℱ}_{2}^{n}$$ (20)

$$\Delta {ℱ}_{2}=\frac{1}{2}{\left[{𝒩}^{n+1}-{𝒩}^{**}\right]}^{2}-\frac{1}{2}{\left[{𝒩}^{n}-{𝒩}^{**}\right]}^{2}$$ (21)

$$=\frac{1}{2}{\left[{𝒩}^{n+1}-{𝒩}^{n}\right]}^{2}+\left[{𝒩}^{n+1}+{𝒩}^{n}-2{𝒩}^{**}\right]$$ (22)

$$=-\frac{1}{2}{\left[{𝒩}^{n+1}-{𝒩}^{n}\right]}^{2}+\left[{𝒩}^{n+1}-{𝒩}^{n}\right]\left[{𝒩}^{n+1}-{𝒩}^{**}\right]$$ (23)

$$\leq \left[{𝒩}^{n+1}-{𝒩}^{n}\right]\left[{𝒩}^{n+1}-{𝒩}^{**}\right].$$ (24)

≤0. (25)

(26)

We can take Π=0 since Ω is an invariant region, giving ${𝒩}^{**}=0$. Thus, ${𝒩}^{n+1}-{𝒩}^{**}>0$, and ${𝒩}^{n+1}-{𝒩}^{n}=\Pi -\theta_{a}\left(S_{a}^{n+1}+M_{a}^{n+1}+R_{t}^{n+1}\right)<0$. This completes the proof. ◻

## 6 Experimental results and discussion

In this section, which includes results and a discussion with recommendations, we will test the validity of our proposed model and simulate it with different tools. We will discuss the obtained results from the model, including the reproduction number and prediction of the nodes in each compartment. We will also test the control strategy that targets firewall protection.

Table 5 compares several related works based on Essential Elements (EE): Firewall (EE1), two populations considered (EE2), Sensitivity Analysis (EE3), and Recommendation Policy (EE4). None of the reviewed works incorporate a recommendation policy (EE4), highlighting a gap in the literature. This analysis underscores the need for future research to integrate recommendation policies into IoT attack propagation and mitigation models.

**Table 5:** Comparison of related works based on Essential Elements (EE).

Prediction Model	EE1	EE2	EE3	EE4
[4]	×	×	✓	×
[35]	×	✓	✓	×
[36]	×	✓	✓	×
[37]	×	✓	×	×
[38]	×	✓	✓	×
Proposed Model	✓	✓	✓	✓

### 6.1 Model implementation

In this subsection, we prepare the model parameters for simulation and validation. First, we set up the simulation environment using the tools listed in Table 6.

**Table 6:** Tools used in this study.

Tool	Description	Usage
gekko 1.0.6	GEKKO is a Python package for machine learning and optimization, specializing in dynamic optimization	Model Predictive Control (MPC)
ode45 - Matlab package	Solve nonstiff differential equations	Proposed model simulation
scipy 1.11.3	Python package for statistics, optimization, and ODE solvers	Used for computing PRCC
networkx 3.1	Python package for the creation, manipulation, and study of complex networks	Modeling IoT network topologies and connectivity

From SciPy 1.11.3, we use RK45 and ‘solve_ivp’ for solver configurations and tune parameters affecting the reproduction number, such as attack and recovery rates. The parameters can be derived from online datasets. By leveraging real-world datasets CICAPTIIoT2024 [22] and UNSW-NB15 [23], we estimate key model parameters, including attack rates ($\beta_{a},\beta_{t}$), recovery rates ($\sigma_{t}$), protection rates (*p*), and removal rates ($\theta_{a},\theta_{t}$). The following mathematical formulas allow for the systematic computation of these parameters:

- $\beta_{a}$, is the ratio of the number of attack packets to the total number of packets multiplied by time.
- $\beta_{t}$, is the ratio of the number of attack packets originating from compromised devices to the total number of packets multiplied by time.
- $\sigma_{t}$, is the reciprocal of the average recovery time.
- *p*, is defined as the ratio of protected devices to the total number of compromised devices multiplied by time.
- $\theta_{a}$, is defined as the reciprocal of the average active time of attackers.
- $\theta_{t}$, is defined as the reciprocal of the average active time of compromised devices.

The proposed model is scalable for complex IoT networks, dynamically adapting to growth via the arrival rate parameter Π. It also supports hierarchical topologies, optimizing communication and propagation.

**Fig 2:**
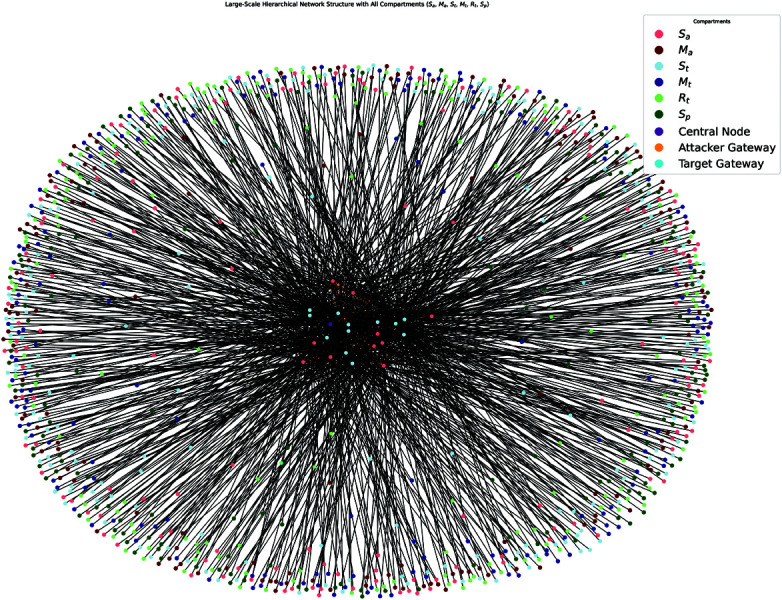
Simulated network for 2000 devices.

**Fig 3:**
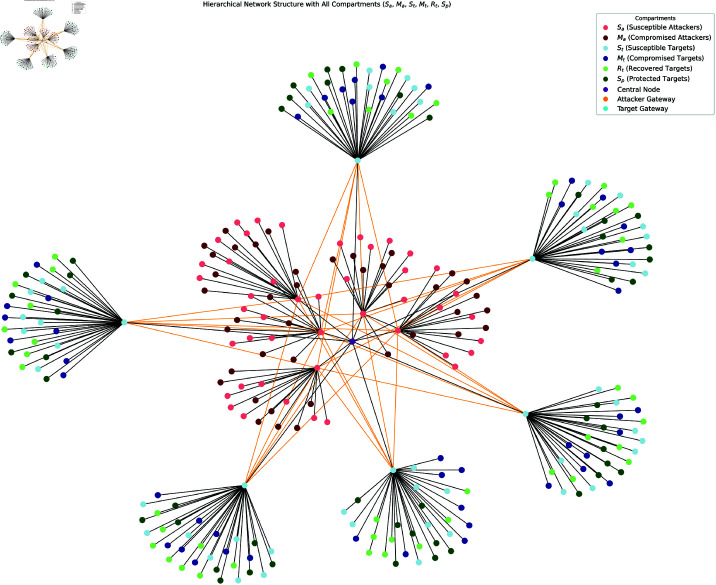
Simulated network for interactions between attackers and targets (330 devices).

Simulations demonstrate that the model can handle large-scale (Fig 2) networks with 2000 of nodes. Moreover, we scaled the network to include 10 attacker clusters, 12 target clusters, and 15 devices per cluster, resulting in 330 devices (plus gateways and the central node) (Fig 3). The model remained computationally feasible and provided accurate predictions even at this scale.

Orange edges represent interactions between attackers and targets, modeled by the equation:


$$\lambda =\beta_{a}M_{a}+\beta_{t}M_{t}$$

This simulation uses a machine with a 12 core CPU and 32 GB RAM. Running time varies from 30 seconds for 330 nodes to 10−−−−13 minutes for 2,000 nodes. The computational complexity of the model is determined by the number of IoT devices and their interactions, with key processes such as infection, recovery, and protection operating at O(N) complexity. In a fully connected network, where every device interacts with all others, the worst-case complexity reaches *O*(*N*^2^) due to pairwise interactions. However, a sparse network assumption significantly improves efficiency by modeling IoT devices as a graph, where each node interacts with only a limited number of neighbors, reducing the complexity to O(kN), where *k* is the average number of connections per device. This approach ensures the model remains scalable for large-scale IoT networks.

### 6.2 Model simulation

In this subsection, we present the simulation framework used to evaluate the proposed model. We employ Monte Carlo simulations to capture randomness and variability in attack dynamics, conduct sensitivity analysis to identify key parameters, and compare stochastic and deterministic solutions. These analyses provide insights into the transient and steady-state behaviors of attackers and IoT devices, validating the model’s robustness and applicability to real-world IoT networks.

The algorithm (see S1 Appendix) is concise and compact, preserving all essential steps while emphasizing the Monte Carlo approach. It highlights the stochastic nature of the simulation with clear annotations such as “Random time step” and “Random event selection.”

**Fig 4:**
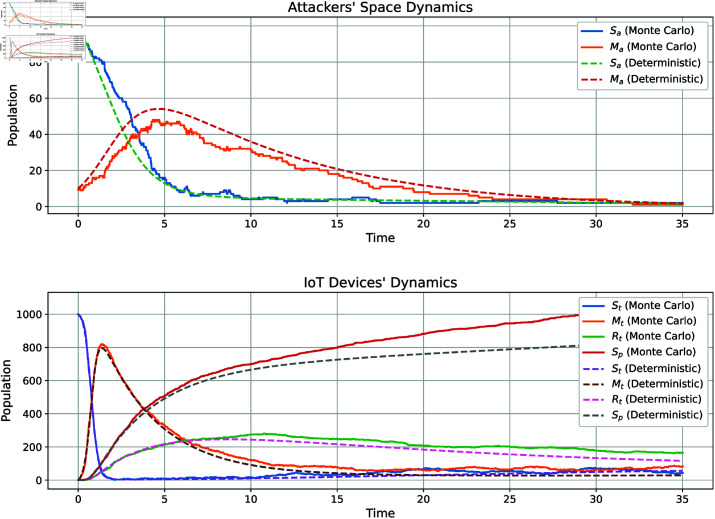
Comparison of stochastic (Monte Carlo) and deterministic solutions for the dynamics of attackers and IoT devices.

To evaluate the robustness of the model under uncertainties, such as uncertain attack rates and varying device security levels, we conducted a comparative analysis using both stochastic (Monte Carlo) and deterministic approaches. Fig 4 illustrates the dynamics of the system involving attackers and IoT devices. The top subplot shows the attackers’ space, where susceptible attackers (*S*_*a*_) transition to malicious attackers (*M*_*a*_). The Monte Carlo simulation captures fluctuations due to randomness, while the deterministic solution provides a smooth average, reflecting the expected behavior. The bottom subplot depicts the dynamics of IoT devices, including susceptible (*S*_*t*_), compromised (*M*_*t*_), recovered (*R*_*t*_), and protected (*S*_*p*_) devices. Here, the Monte Carlo results exhibit step-like behavior due to stochastic effects, whereas the deterministic solution offers a continuous approximation. Both approaches reveal a transient phase followed by a steady state, demonstrating the system’s balance between infection, recovery, and protection. This analysis highlights the importance of stochastic modeling for capturing variability under uncertainties and deterministic modeling for understanding average behavior. These insights are critical for developing robust mitigation strategies, such as improving recovery and protection mechanisms, to address DDoS attacks in IoT networks with varying levels of device security and attack rates.

**Fig 5:**
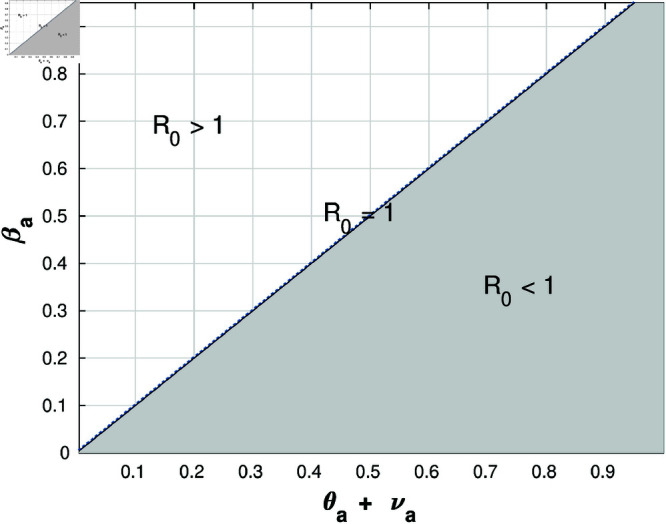
The reproduction number of attacking population analysis.

**Fig 6:**
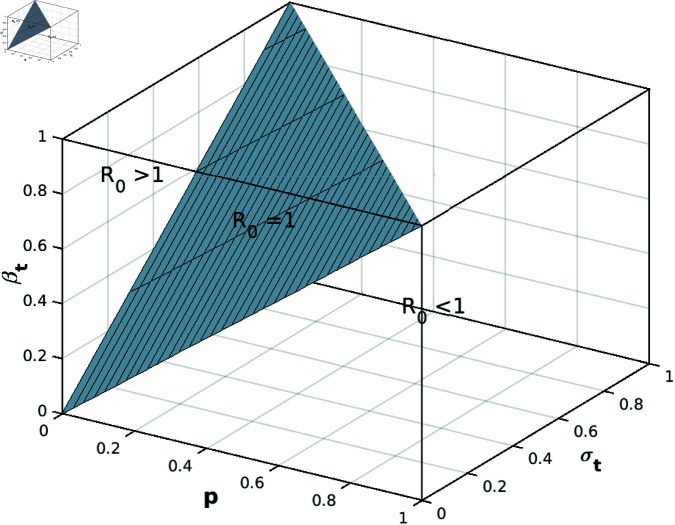
The reproduction number *R*_*Target*_ of targeted population analysis.

Figures 5 and 6 elucidate the dynamics of reproduction numbers for both attacking and targeted populations. This analysis facilitates discerning instances of successful and unsuccessful attacks.

Fig 5 shows the variation of the attack reproduction number, ${ℛ}_{\text{attack}}$, with respect to the variables $\beta_{a}\;\theta_{a}$. The lower triangle of the graph represents the region where the attack fails, i.e., the infection becomes endemic. The upper triangle represents the region where the attack is successful, i.e., the infection is eradicated. The line ${ℛ}_{\text{attack}}=1$ separates these two regions. In other words, if the reproduction number of an attack is greater than one, then each infected node will infect more than one other node, leading to an exponential increase in the number of infected nodes. This makes it difficult to stop the attack and increases its chances of succeeding. Also, it shows that when the reproduction number is less than one, an endemic case appears, and the attack will be prevented due to the decreasing number of attacking nodes. In other words, if the reproduction number of an attack is less than one, each infected node will not infect more than one other node, leading to a collapse in the number of infected nodes. This makes it easier to stop the attack. The analogous conclusion applies to Fig 6.

**Fig 7:**
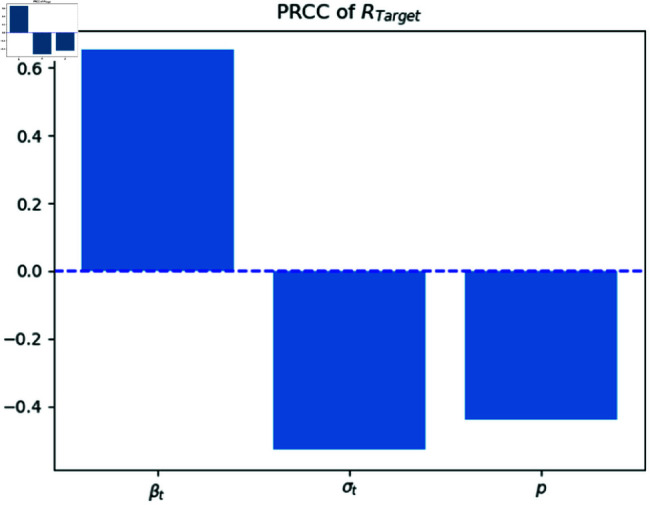
PRCC elasticity analysis of $R_{0}^{Target}$.

**Fig 8:**
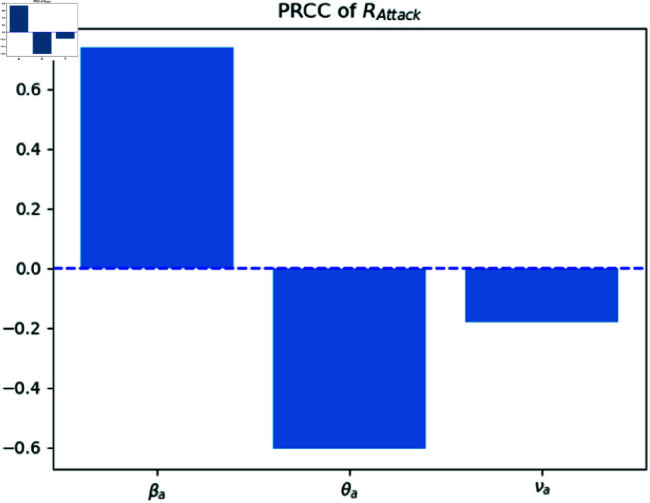
PRCC of $R_{0}^{a}$ analysis.

The Partial Rank Correlation Coefficient (PRCC) is a valuable metric for assessing the sensitivity of the basic reproduction number (Fig 7). PRCC values, expressed as percentages, provide insights into the strength and directionality of correlation between each epidemiological parameter ($\beta_{a},\;\sigma_{a}$ and *p*) and the basic reproduction number *R*_*target*_. These coefficients quantify the extent to which changes in a specific parameter impact the equilibrium dynamics of infection. A positive PRCC ($\beta_{a}$) indicates a direct relationship, where an increase in the parameter positively influences the reproduction number. Conversely, a negative PRCC ($\sigma_{a}$ and *p*) signifies an inverse relationship, where higher parameter values decrease the reproduction number. These findings are crucial for understanding the sensitivity of disease transmission dynamics and inform effective intervention strategies. The findings of Fig 8 can be concluded as the previous figure. The weak relation of *R*_*attack*_ and $\nu_{a}$ is because of the lower recovery rate in the attacking population.

**Fig 9:**
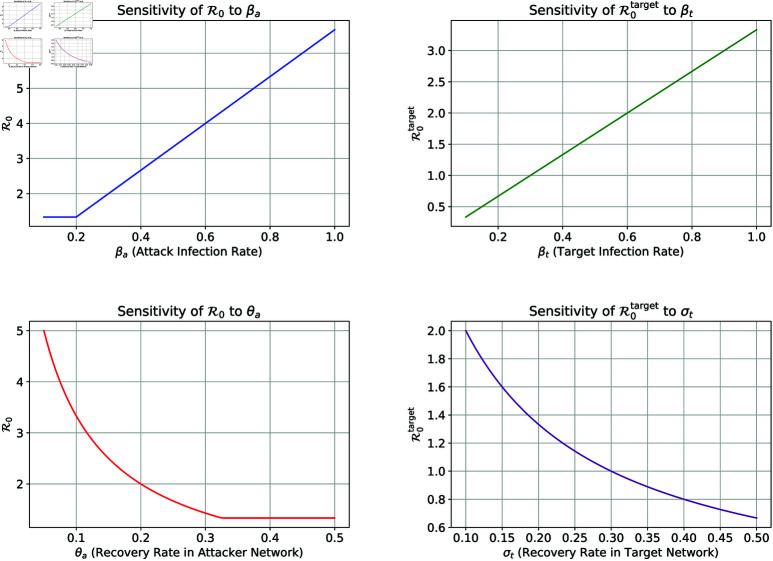
Sensitivity analysis of the reproduction number *R*_0_ to key parameters.

The left plots in Fig 9 illustrate the impact of $\beta_{a}$ and $\theta_{a}$ on *R*_0_. However, the right plots show the influence of $\beta_{t}$ and $\sigma_{t}$ on *R*_0_. These plots highlight the relative sensitivity of *R*_0_ to each parameter, providing insights into their roles in the spread of DDoS attacks and guiding effective control strategies.

**Fig 10:**
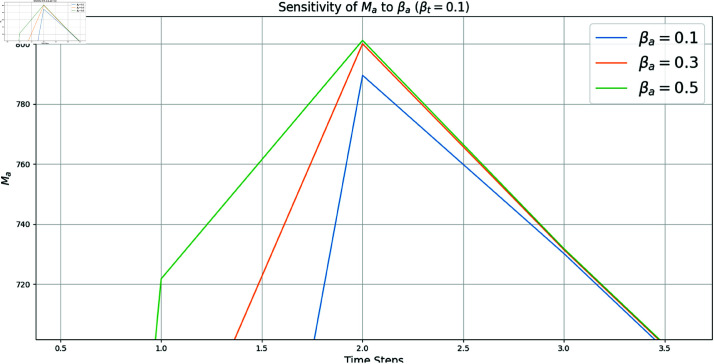
Sensitivity analysis of *M*_*a*_ (malicious attackers) to $\beta_{a}$.

Fig 10 demonstrates how variations in $\beta_{a}$ influence the number of malicious attackers over time, highlighting the critical role of the attack rate in the propagation of DDoS attacks. This analysis provides insights into the effectiveness of strategies targeting $\beta_{a}$ to mitigate the spread of malicious activity in IoT networks.

**Fig 11:**
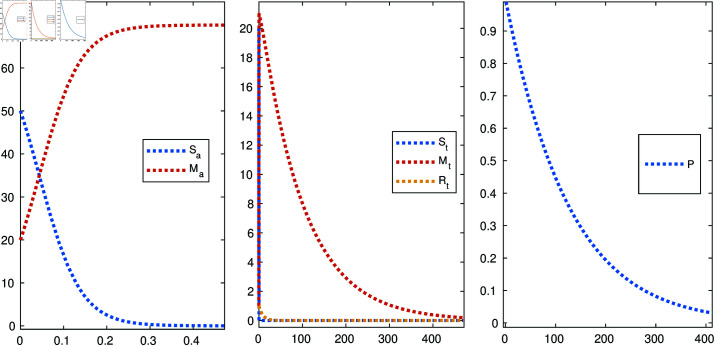
*R*_0_>1 compartment analysis when we have successful attack.

Fig 11 shows a graph of the percentage of successful attacks when the attacker has successfully attacked the target. The graph shows three curves, one for each of the three values of *R*_0_>1. The graph shows that the percentage of successful attack reproduction number is less than 1 in the case of a failed cyberattack. The network’s defense graph also shows that the percentage of successful attacks decreases as the number of nodes increases. This is because there are more opportunities for the infection to be stopped as the number of nodes increases. The dashed line in the graph represents the percentage of successful attacks if the attacker does not target any nodes. This line is at 50%, which means that the attacker is equally likely to succeed or fail regardless of the *R*_0_>1 value or the number of nodes.

**Fig 12:**
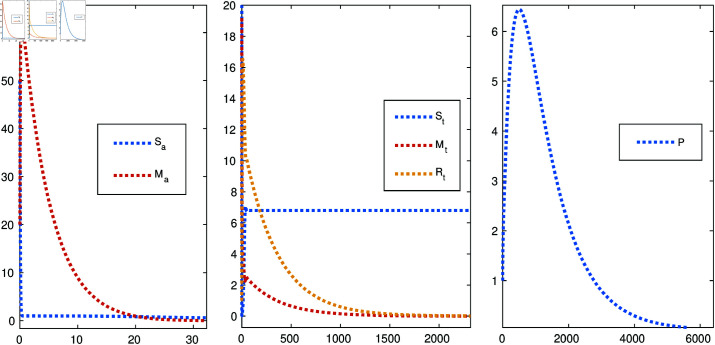
*R*_0_<1 compartment analysis when the cyberattack failed.

Fig 12 shows the curve for the case where the cyberattack failed, called the extinction curve. It is characterized by a reproduction number (*R*_0_) less than 1. This means that, on average, each infected node infects less than one other node. As a result, the number of infected nodes gradually decreases and eventually reaches zero. The reproduction number is less than 1 in the case of a failed cyberattack because the network’s defenses can stop the spread of the infection. These defenses can include firewalls, intrusion detection systems, and antivirus software. They can also include human factors, such as employee training and awareness. In addition to the network’s defenses, the failure of a cyberattack can also be due to other factors, such as the quality of the attack code, the attacker’s resources, and the attack’s timing.

**Fig 13:**
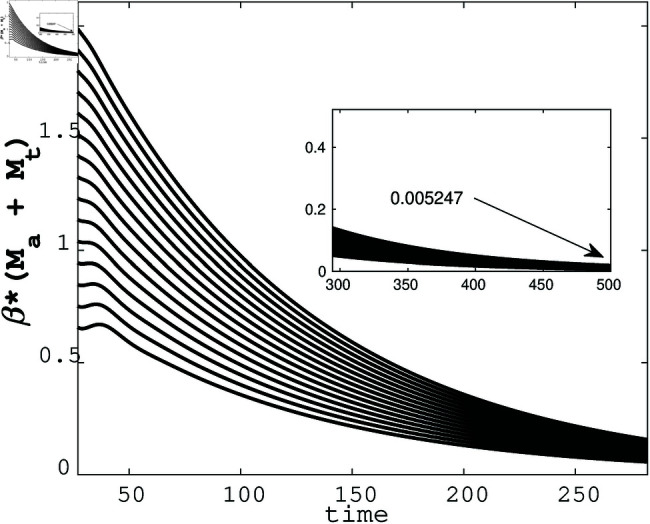
The proportion of compromised devices during a cyber IoT attack.

Fig 13 shows a DDoS attack is a cyberattack that attempts to overload a target with traffic, making it unavailable to legitimate users. The percentage of infected devices in a DDoS attack typically starts high and decreases over time. This is because the attack initially successfully overwhelms the target, but the target’s defenses eventually start to mitigate the attack. The initial high percentage of infected devices is because the attack can quickly infect many devices. This is normally achieved by leveraging vulnerabilities or social engineering, such as tricking users into accessing a malicious webpage or other entry attack vectors. Throughout the attack, the defenses of the target weaken the attack. This would take the form of blocking the volumes of malicious traffic, filtering out the infected devices, or even strengthening the target’s infrastructure. This causes the percentage of infected devices to decrease over time until they eventually reach a stable state.

### 6.3 Designing the optimal control strategy

The formulation of efficient preventive measures is very cautious in cybersecurity. In particular, we suggest an optimal control strategy for preventing firewall attacks. This strategy mitigates the effect of those unexpected attacks by judiciously varying parameters associated with the firewall’s configuration and response. The prevention factor of the firewall, which is represented as *f*_*p*_, will be used in reconstructing the prime model as follows:

$${\dot{S}}_{t}=\Pi -(1-f_{p})(\beta_{a}M_{a}+\beta_{t}M_{t})S_{t},\;\dot{M_{t}}=(1-f_{p})(\beta_{a}M_{a}+\beta_{t}M_{t})S_{t}-\sigma_{t}M_{t}-pM_{t},\;{\dot{R}}_{t}=\sigma_{t}M_{t}-\theta_{t}R_{t}\;{\dot{S}}_{p}=pM_{t}$$ (27)

By examining the effect of these parameters on the success or failure of such an attack, we can better personalize our defense strategies. Firewalls, as they turned out to be the first line of defense against cyberattacks, are very important in maintaining the integrity of the network. Next-generation firewalls have threat prevention capabilities that allow for early detection and blocking of attempted attacks before they breach the corporate network.

The below steps are suggested to improve their effectiveness: Parameter Analysis: Some vital parameters about the firewall configuration will be studied, like transmission rates, access controls, filtering rules, etc. These parameters significantly impact the effectiveness of prevention against different kinds of attacks. Optimized Control Strategy: Mathematical modeling and optimization methodology will yield time-varying and cost-effective solutions for malware outbreak mitigation.

For instance, strategies like quarantine and vaccination should be promptly implemented at the onset of an attack, while continuous monitoring and patch application remain essential [39]. In addition, the basic reproduction number (a threshold value governing malware diffusion) is used to quantify how adjustments in firewall parameters influence attack propagation. The new reproduction number is clearly defined as


$${ℛ}_{0}=(1-f_{p})\times \max\{{ℛ}_{\text{attack}},\;{ℛ}_{\text{Target}}\}$$

**Fig 14:**
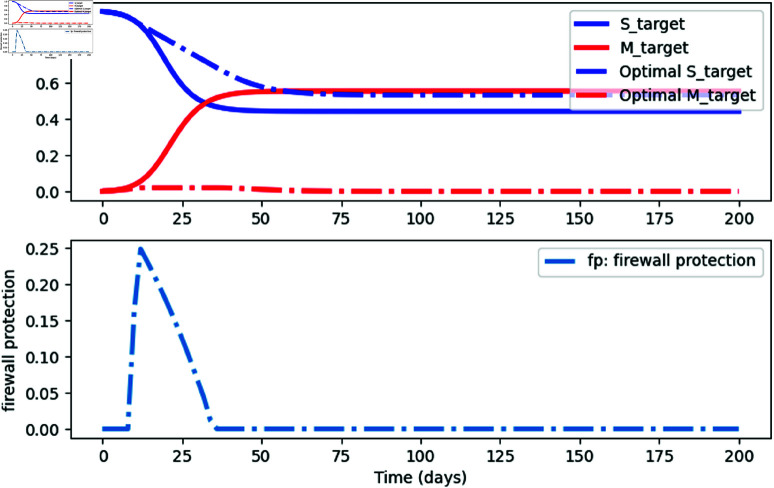
An optimal control strategy for firewall protection *f*_*p*_

Applying the *gekko* Python tools, we get the simulation in Fig 14. We observed a transition from attack success to failure. Notably, the firewall efficiency did not surpass 0.25, indicating that achieving optimal protection requires further enhancements.

Based on this simulation, we can define the policy based on the following recommendations.

- Deploy Firewalls: Install firewalls on all critical nodes to reduce infection spread.
- Optimize Firewall Efficiency: Maintain a protection rate of at least 0.125 to mitigate attacks effectively.
- Educate Stakeholders: Raise awareness about firewall benefits and their role in network security.

These steps enhance resilience, reduce infections, and maintain network integrity.

Now, we will define the optimization problem, which aims to minimize the impact of malicious attackers (*M*_*a*_) and compromised devices (*M*_*t*_), while maximizing the number of protected (*S*_*p*_) and recovered (*R*_*t*_) devices. This will reduce the cost of maintaining the network and prevent IoT devices from being lost. The objective function is defined as:


$$J=\int_{0}^{T}\left(w_{1}M_{a}(t)+w_{2}M_{t}(t)-w_{3}S_{p}(t)-w_{4}R_{t}(t)\right)dt,$$

where $w_{1},w_{2},w_{3},w_{4}$ are weights representing the importance of each term, and *T* is the time horizon.

Control variables are introduced to influence the system dynamics: *u*_1_(*t*) reduces the attack rate ($\beta_{a}$), *u*_2_(*t*) increases the recovery rate ($\sigma_{t}$), and *u*_3_(*t*) increases the protection rate (*p*). These control variables are bounded as:


$$0\leq u_{1}(t)\leq u_{1,\max},\;0\leq u_{2}(t)\leq u_{2,\max},\;0\leq u_{3}(t)\leq u_{3,\max}.$$

The system dynamics are modified to incorporate the control variables:


$$S_{a}^{n+1}-S_{a}^{n}=-\beta_{a}(1-u_{1})S_{a}^{n+1}M_{a}^{n+1}-\theta_{a}S_{a}^{n+1}+\nu_{a}M_{a}^{n+1},$$

$$M_{a}^{n+1}-M_{a}^{n}=\beta_{a}(1-u_{1})S_{a}^{n+1}M_{a}^{n+1}-(\theta_{a}+\nu_{a})M_{a}^{n+1},$$

$$S_{t}^{n+1}-S_{t}^{n}=\Pi -\left(\beta_{a}(1-u_{1})M_{a}^{n+1}+\beta_{t}M_{t}^{n+1}\right)S_{t}^{n+1},$$

$$M_{t}^{n+1}-M_{t}^{n}=\left(\beta_{a}(1-u_{1})M_{a}^{n+1}+\beta_{t}M_{t}^{n+1}\right)S_{t}^{n+1}-(\sigma_{t}+u_{2})M_{t}^{n+1}-(p+u_{3})M_{t}^{n+1},$$

$$R_{t}^{n+1}-R_{t}^{n}=(\sigma_{t}+u_{2})M_{t}^{n+1}-\theta_{t}R_{t}^{n+1},$$

$$S_{p}^{n+1}-S_{p}^{n}=(p+u_{3})M_{t}^{n+1}.$$

The objective function *J* balances the trade-off between minimizing malicious activities (*M*_*a*_ and *M*_*t*_) and maximizing protective measures (*S*_*p*_ and *R*_*t*_). The control variables $u_{1},u_{2},u_{3}$ represent efforts to reduce attacks, increase recovery, and enhance protection. The modified dynamics incorporate these controls, such as reducing the attack rate by $\beta_{a}(1-u_{1})$, increasing the recovery rate by $\sigma_{t}+u_{2}$, and increasing the protection rate by *p* + *u*_3_.

**Fig 15:**
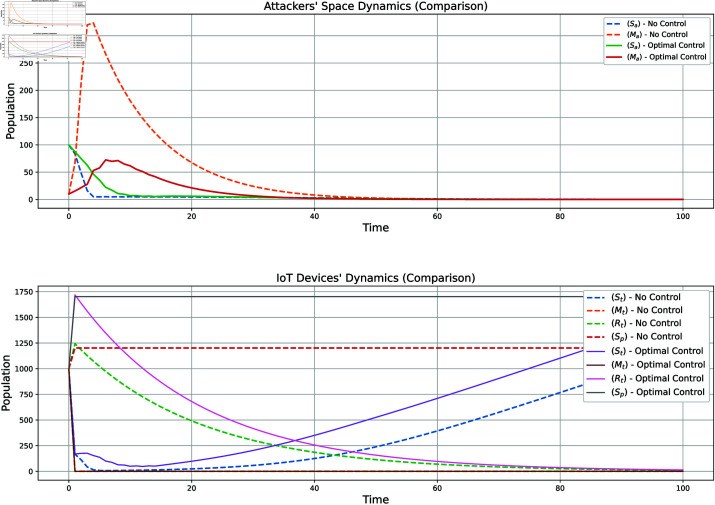
Comparison of attacker and target populations with and without control strategy.

Fig 15 illustrates the impact of implementing an optimal control strategy on the dynamics of attacker and target populations. By effectively reducing the number of attackers and targets over time, the approach enhances network security and minimizes the associated costs of mitigation and recovery. This demonstrates the dual benefit of the control strategy: improving security while optimizing resource expenditure.

### 6.4 Model integration

In this section, we propose an application of our model for integration with a cyber threat intelligence platform. The API facilitates communication between the CTI-platform (Cyber Threat Intelligence Application) and the Model Plugin. The CTI-App provides real-time data about the IoT network’s compartments, time, and risk level of malware. At the same time, the Model Plugin processes this data and returns predictions and recommendations (see S2 Appendix.).

The proposed API structure delivers significant advantages, including real-time predictions powered by the latest threat data, ensuring timely insights into attack propagation. To ensure the confidentiality and integrity of data exchanged between the CTI-App and the Model Plugin, all API requests and responses will be encrypted using TLS/SSL to prevent unauthorized access during transmission. It offers actionable recommendations, enabling stakeholders to proactively address threats, and is scalable to handle large IoT networks while adapting to evolving risks. Its seamless integration with existing cybersecurity tools enhances practicality and usability in real-world scenarios.

The system includes an auto-connecting interface with cyber intelligence applications, ensuring effortless integration and requiring no user intervention beyond monitoring results. Providing clear, actionable recommendations simplifies decision-making and prioritizes stakeholder input, creating a user-friendly and efficient experience.

The proposed model for DDoS attack propagation in IoT networks has several limitations, including the lack of consideration for power consumption, other malware types, and complex network topologies. Additionally, the model does not explicitly address computational constraints, such as runtime scalability for large-scale networks. Future work should extend the model to incorporate energy-aware metrics, diverse malware behaviors (e.g., ransomware, botnets), and advanced topologies (e.g., hierarchical, mesh). Moreover, optimizing computational efficiency and exploring parallel processing techniques will be crucial for handling large-scale simulations. The model should also account for device heterogeneity and dynamic parameter adaptation to better reflect real-world scenarios. Combining multiple malware types and integrating real-time threat data will enhance its predictive capabilities and practical relevance.

A roadmap for future work includes integrating network topology parameters to model realistic IoT connectivity and incorporating resource constraints to account for processing power and energy consumption. Additionally, the model should adopt adaptive security strategies that dynamically adjust policies based on network size and attack severity. These enhancements will improve scalability, computational efficiency, and real-world applicability, enabling robust mitigation strategies and proactive IoT security measures.

## Conclusion

This study investigates the propagation dynamics and mitigation strategies of DDoS attacks in IoT networks. We have quantified key factors influencing the spread of such attacks by analyzing the sensitivity of the reproduction number, attacker behavior, and compartmental proportions. Our findings highlight the importance of a multi-faceted approach to securing IoT devices and networks. To enhance the robustness of our analysis, we integrated statistical Monte Carlo simulations, which provide probabilistic insights into attack propagation and the effectiveness of mitigation strategies under varying network conditions. This allows for a more comprehensive evaluation of uncertainties in real-world scenarios. Furthermore, we explored optimal control techniques to identify strategies that minimize the impact of DDoS attacks while maintaining network functionality. Sensitivity analysis revealed the most critical parameters influencing attack spread and mitigation effectiveness. Our proposed mitigation strategies include strengthening IoT security through regular updates, strong authentication protocols, and enabling advanced security features. Raising user awareness about DDoS threats is essential to prevent devices from being exploited in attacks. Additionally, leveraging automated DDoS mitigation tools to filter malicious traffic and implementing cooperative threat intelligence sharing among organizations can improve early detection and response strategies. This study contributes to the ongoing efforts to enhance IoT security by integrating mathematical modeling with computational simulations and real-world applicability. We hope our findings aid in developing effective, data-driven mitigation frameworks to counteract the growing threat of DDoS attacks.

## Supporting information

S1 Appendix Monte Carlo Simulation for Attackers and IoT Devices
(PDF)

S2 Appendix The CTI-App sends a request to the Model Plugin with the following structure.
(PDF)
