# Correction: Mathematical analysis of the dynamics of cyberattack propagation in IoT networks

**DOI:** 10.1371/journal.pone.0339507

**Published:** 2025-12-22

**Authors:** The PLOS One Staff

The Acknowledgement section for this article is missing. The Acknowledgement statement is: This study was supported by the Princess Nourah bint Abdulrahman University Researchers (Grant No. PNURSP2025R518), Princess Nourah bint Abdulrahman University, Riyadh, Saudi Arabia

The publisher apologizes for the error.
