# Supplementary material for: Mathematical analysis of the dynamics of cyberattack propagation in IoT networks
Source: PLoS One. 2025 May 16;20(5):e0322391. doi: 10.1371/journal.pone.0322391 (PMC12083842; doi:10.1371/journal.pone.0322391)
Supplement: S1 Appendix — Monte Carlo Simulation for Attackers and IoT Devices (PDF) [file pone.0322391.s001.pdf]

Supporting information

Algorithm 1 Monte Carlo Simulation for Attackers and IoT Devices

Monte Carlo Method: Simulate system dynamics using stochastic events and random sampling.
Input: Parameters $(\beta_a, \beta_t, \theta_a, \nu_a, \sigma_t, p, \theta_t, \Pi)$, initial populations $(S_a, M_a, S_t, M_t, R_t, S_p)$, $t_{\max}$
Output: $\textit{time\_points}, \textit{states}$
Initialize $\textit{time} \leftarrow 0$, $\textit{time\_points} \leftarrow [\textit{time}]$, $\textit{states} \leftarrow [(S_a, M_a, S_t, M_t, R_t, S_p)]$
while $\textit{time} < t_{\max}$ **do**
    Compute $\lambda \leftarrow \beta_a \cdot M_a + \beta_t \cdot M_t$
    Compute $\textit{rates} \leftarrow [\beta_a \cdot S_a \cdot M_a, \theta_a \cdot S_a, \nu_a \cdot M_a, \theta_a \cdot M_a, \lambda \cdot S_t, \sigma_t \cdot M_t, p \cdot M_t, \theta_t \cdot R_t, \Pi]$
    $\textit{total\_rate} \leftarrow \sum(\textit{rates})$
    if $\textit{total\_rate} == 0$ **then**
        Break
    end if
    $dt \leftarrow \text{Exponential}(1/\textit{total\_rate})$ {Random time step}
    $\textit{time} \leftarrow \textit{time} + dt$
    $\textit{event} \leftarrow \text{RandomChoice}(\textit{rates}/\textit{total\_rate})$ {Random event selection}
    Update $(S_a, M_a, S_t, M_t, R_t, S_p)$ based on *event*
    Append *time* to *time_points* and $(S_a, M_a, S_t, M_t, R_t, S_p)$ to *states*
end while
Return $\textit{time\_points}, \textit{states}$

S1 Appendix.
