# Supplementary material for: Mathematical analysis of the dynamics of cyberattack propagation in IoT networks
Source: PLoS One. 2025 May 16;20(5):e0322391. doi: 10.1371/journal.pone.0322391 (PMC12083842; doi:10.1371/journal.pone.0322391)
Supplement: S2 Appendix — The CTI-App sends a request to the Model Plugin with the following structure. (PDF) [file pone.0322391.s002.pdf]

S2 Appendix. The CTI-App sends a request to the Model Plugin with the following structure:


```
{
  "timestamp": "2025-00-00T00:00:00Z",  // Timestamp of the data
  "compartments": {
    "S_a": 1000, "M_a": 50, "S_t": 5000, "M_t": 10, "R_t": 200,
    "S_p": 100  },
  "risk_level": "high",  // Risk level of malware (low, medium, high)}
```


The Model Plugin processes the request and returns predictions and recommendations in the following structure:


```
{
  "predictions": [
    {
      "time": "2025-10-15T13:00:00Z",  // Timestamp of the prediction
      "S_a": 950,  // Predicted
      "M_a": 100,  // Predicted
      "S_t": 4800, // Predicted
      "M_t": 150,  // Predicted
      "R_t": 250,  // Predicted
      "S_p": 120   // Predicted
    },
    {
      "time": "2023-10-15T14:00:00Z",
      "S_a": 900,
      "M_a": 150,
      "S_t": 4600,
      "M_t": 200,
      "R_t": 300,
      "S_p": 140
    },
    // Additional predictions for each time step...
  ],
  "recommendations": [
    {
      "action": "block_malicious_ips",  // Recommended action
      "priority": "high",               // Priority of the action
      "description": "Block IP addresses
      associated with the attacker’s network."
    },
    {
      "action": "isolate_compromised_devices",
      "priority": "medium",
      "description": "Isolate devices identified as compromised."
    },
    {
      "action": "increase_network_monitoring",
      "priority": "low",
      "description": "Increase monitoring of network traffic."
    }
  ]
}
```
